# Fasting plasma glucose 6–12 weeks after starting insulin glargine predicts likelihood of treatment success: a pooled analysis

**DOI:** 10.1111/j.1464-5491.2012.03640.x

**Published:** 2012-07

**Authors:** D Karl, R Zhou, A Vlajnic, M Riddle

**Affiliations:** 1 The Endocrine ClinicPortland, OR; 2 Medpace Inc.Cincinnati, OH; 3 Sanofi USBridgewater, NJ; 4 Oregon Health and Science UniversityPortland, OR, USA

**Keywords:** fasting plasma glucose, glycated haemoglobin, HbA_1c_, insulin glargine, Type 2 diabetes

## Abstract

**Aims:**

To evaluate whether fasting plasma glucose values measured early during insulin therapy can identify patients with Type 2 diabetes who may not achieve adequate glycaemic control after 6 months and will require additional treatment.

**Methods:**

Patient-level data from seven prospective, randomized, controlled studies using treat-to-target methods were pooled to evaluate the efficacy of insulin glargine. Fasting plasma glucose was measured at baseline, week 6 or 8 (6/8) and week 12. HbA_1c_ was measured at week 24 to assess glycaemic control.

**Results:**

One thousand and thirty-six patients (56% male, 81% white) were included in the analysis (mean age 56.3 years; duration of diabetes 8.4 years). Baseline mean fasting plasma glucose was 11.2 mmol/l and mean HbA_1c_ was 73 mmol/mol (8.8%). After 24 weeks of treatment, mean HbA_1c_ decreased to 53 mmol/mol (7.0%); 56% of patients reached a target HbA_1c_≤ 53 mmol/mol (7.0%). Significant correlations with week 24 HbA_1c_ were obtained for fasting plasma glucose measured at week 6/8 and week 12 (*r* = 0.32; *P* < 0.0001 for both). Patients with fasting plasma glucose > 10 mmol/l at week 6/8 or week 12 were significantly less likely to achieve the HbA_1c_ target at the end of treatment than patients with fasting plasma glucose < 8.9 mmol/l (*P* < 0.0001 for both). If fasting plasma glucose was > 10 mmol/l at week 6/8 or week 12, patients had only a 27% chance of reaching the HbA_1c_ goal.

**Conclusions:**

Fasting plasma glucose remaining > 10 mmol/l after 6–12 weeks of glargine therapy indicates that reaching target HbA_1c_≤ 53 mmol/mol (7.0%) is unlikely and calls for individualized attention to consider further therapeutic options.

## Introduction

Many patients treated with lifestyle modifications and oral anti-diabetic drugs eventually require insulin therapy [1]. Adding titrated insulin glargine to the drugs reduces HbA_1c_ to < 53 mmol/mol (7.0%) for many patients, but some require additional therapy [2,3]. Early identification of patients for whom glargine and oral anti-diabetic drugs may not be sufficient would be helpful.

Concurrent measurements of fasting plasma glucose and HbA_1c_ levels during stable therapy are strongly correlated, [4,5]. However, when treatment is intensified, fasting plasma glucose values improve immediately, whereas HbA_1c_ values lag behind, not reaching a stable new value for 4 months or more. Therefore, fasting plasma glucose measured at early follow-up visits might predict future success in reaching HbA_1c_ targets. To test this concept, we performed a pooled analysis of patient-level data obtained in studies in which treatment with glargine was started using similar treat-to-target methods.

## Patients and methods

### Study selection

Sixty-three studies in adults with Type 2 diabetes completed between 1997 and 2007 were evaluated. For inclusion in the pooled analysis, a study had to have a prospective, randomized, controlled design conducted according to Good Clinical Practice standards; using basal insulin therapy with glargine (but without prandial insulin) added to lifestyle alone or stable oral anti-diabetic drug therapy, and a treat-to-target strategy with a predefined insulin titration algorithm targeting fasting plasma glucose ≤ 5.6 mmol/l; and including fasting plasma glucose measurements at baseline, week 6 or 8 (6/8) and week 12 as well as systematic collection of reports of hypoglycaemia. Seven studies met these criteria [2,6–11] (data on file; Sanofi US, Bridgewater, NJ, USA). For studies with longer duration, only data from the first 24 weeks were used. Participants were eligible for analysis if fasting plasma glucose values at baseline, week 6/8 and week 12 and HbA_1c_ measurements at week 24 were all available. Patient level data for 1036 participants were included.

### Outcome measures

Laboratory measurements of fasting plasma glucose from samples collected at study sites were divided into three categories: < 8.9, ≥ 8.9 to < 10 or ≥ 10 mmol/l. Correlations between study site fasting plasma glucose values and those obtained at home by patient self-measurement were determined. Treatment was considered successful if HbA_1c_ at week 24 was ≤ 53 mmol/mol (7.0%). Symptomatic hypoglycaemia was defined as all events reported with symptoms. Glucose-confirmed symptomatic hypoglycaemia included events with concurrent glucose < 2.8 mmol/l. Severe hypoglycaemia comprised all symptomatic events for which the patient required assistance and had either a blood glucose level < 2 mmol/l or prompt recovery after oral carbohydrate, intravenous glucose or glucagon administration.

### Statistical analysis

Summary statistics were calculated for HbA_1c_ at baseline and week 24. Independent analyses of fasting plasma glucose values were performed for the correlation between week 6/8 fasting plasma glucose and week 24 HbA_1c_, and the correlation between week 12 fasting plasma glucose and week 24 HbA_1c_. For each, week 24 HbA_1c_ and change in HbA_1c_ from baseline to week 24 were analysed continuously using an analysis of covariance model, with study as a factor and post-baseline visit fasting plasma glucose as a covariate. Week 24 HbA_1c_ was dichotomized with ≤ 53 mmol/mol (7.0%) as a target threshold. Additional analyses were explored by adding baseline fasting plasma glucose as a covariate, or using categorized fasting plasma glucose instead of the measurement as a continuous variable, as well as using dichotomized week 24 HbA_1c_ value as the response variable. The general association between success of reaching target HbA_1c_ and each fasting plasma glucose category was assessed using a Cochran–Mantel–Haenszel statistic. To verify the relevance of central laboratory fasting plasma glucose values obtained at visits to home self measurements, a subset of samples (*n* = 490) obtained at visits were compared by Pearson correlation coefficients with plasma referenced, self-measured, fasting capillary blood samples obtained the same week at home.

## Results

### Baseline characteristics and glycaemic responses

The 1036 participants were 56% male and 81% white with mean ± sd age 56.3 ± 9.9 years and duration of diabetes 8.4 ± 5.9 years. Prior (and continued) therapies included 1–3 oral anti-diabetic drugs (metformin, sulphonylurea, thiazolidinedione). Mean HbA_1c_ was 73 mmol/mol (8.84 ± 1.03%) and mean fasting plasma glucose was 11.2 ± 3.0 mmol. Examined by ranges of HbA_1c_ at baseline, 63% of patients had fasting plasma glucose ≥ 10 mmol/l, 13% had fasting plasma glucose from 8.9 to < 10 mmol/l and 24% had fasting plasma glucose < 8.9 mmol/l. Measurements at home (mean ± sd; 8.85 ± 3.39 mmol/l) correlated strongly with laboratory measurements (8.65 ± 3.18 mmol/l; *r* = 0.778; *P* < 0.0001).

Adding glargine to oral anti-diabetic drugs reduced both measures of glycaemic control, but with differing patterns (Table 1). Mean fasting plasma glucose declined to 7.3 mmol/l at 6/8 weeks, 6.8 mmol/l at 12 weeks and 6.7 mmol/l at 24 weeks. Mean HbA_1c_ was 56 mmol/mol (7.3%) at 12 weeks and 53 mmol/mol (7.0%) at 24 weeks, with 56% of patients at or below that level.

**Table 1:** Changes in fasting plasma glucose during treatment

Patients		Baseline	Week 6 or 8*	Week 12	Week 24
All patients, mean (sd) fasting plasma glucose, mmol/l		11.1 (3.0)	7.3 (2.2)	6.8 (2.0)	6.7 (1.9)
All patients, mean (sd) HbA_1c_, mmol/mol†		73 8.84 (1.03)	—	56 7.27 (0.90)	53 7.03 (0.86)
Patients (*n*, %) and glucose [mean (sd), mmol/l] within each fasting plasma glucose group					
Fasting plasma glucose < 8.9 mmol/l	*n*, %	246 (23.7)	827 (79.8)	897 (86.6)	923 (89.1)
	Mean (sd)	7.6 (0.9)	6.5 (1.3)	6.2 (1.3)	6.2 (1.3)
	HbA_1c_, % mean (sd)	8.17 (0.71)	—	7.16 (0.80)	6.95 (0.80)
Fasting plasma glucose 8.9 to < 10 mmol/l	*n*, %	139 (13.4)	107 (10.3)	77 (7.4)	57 (5.5)
	Mean (sd)	9.4 (0.3)	9.4 (0.3)	9.4 (0.3)	9.3 (0.3)
	HbA_1c_, % mean (sd)	8.42 (0.82)	—	7.72 (1.04)	7.42 (0.90)
Fasting plasma glucose ≥ 10 mmol/l	*n*, %	651 (62.8)	102 (9.8)	62 (6.0)	56 (5.4)
	Mean (sd)	12.8 (2.4)	11.8 (1.6)	11.6 (1.4)	11.7 (1.6)
	HbA_1c_, %, mean (sd)	9.18 (1.01)	—	8.25 (1.24)	7.9 (1.14)

* HbA_1c_ was not measured at week 6/8.

† Standard deviation (sd) is not calculable.

### Fasting plasma glucose as a predictor of treatment outcome

The correlation between baseline fasting plasma glucose and week 24 HbA_1c_ considered as continuous variables was weak but statistically significant in this large sample (*r* = 0.169; *P* < 0.0001). The correlation with week 24 HbA_1c_ was stronger for fasting plasma glucose measured at week 6/8 (*r* = 0.319; *P* < 0.0001) and week 12 (*r* = 0.317; *P* < 0.0001).

**FIGURE 1:**
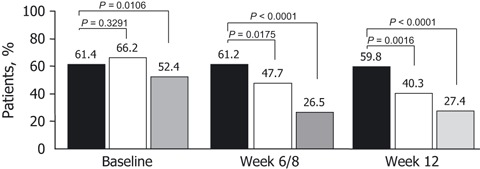
Percentages of patients achieving HbA_1c_≤ 53 mmol/mol (7.0%) at week 24 at three stages of therapy, by range of fasting plasma glucose values at each stage: (▪) < 8.9 mmol/l; (□) 8.9 to < 10 mmol/l; (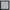) ≥ 10 mmol/l.

When the ability of baseline fasting plasma glucose to predict later attainment of the HbA_1c_ target level was examined by range of fasting plasma glucose, the limited predictive power of this measurement was evident (Fig. 1). Percentages of participants attaining the HbA_1c_ target for those starting with fasting plasma glucose < 8.9, 8.9 to < 10 and ≥ 10 mmol/l were 61, 66 and 52%. However, corresponding percentages at week 6/8 were 61, 48 and 26.5%; at 12 weeks they were 60, 40 and 27%. For the general association between reaching target HbA_1c_ and these fasting plasma glucose categories, *P*-values were 0.001, < 0.0001 and < 0.0001 for the fasting plasma glucose categories at baseline, week 6/8 and week 12, respectively.

Persistence of higher fasting plasma glucose early in therapy was associated with the possibility of having very high HbA_1c_ at 24 weeks. In the group with fasting plasma glucose < 8.9 mmol/l at 12 weeks just 8% of participants had HbA_1c_ above 64 mmol/mol (8.0%) at 24 weeks. In contrast, in the group with fasting plasma glucose ≥ 10 mmol/l at 12 weeks, 39% had week 24 HbA_1c_ above 64 mmol/mol (8.0%), 14.5% above 75 mmol/mol (9.0%) and 6.5% above 86 mmol/mol (10.0%).

### Hypoglycaemia

Severe hypoglycaemia occurred in 1.4% of participants, with 0.05 events per participant-year. Symptomatic hypoglycaemia was reported by 65% of participants with 12-week fasting plasma glucose < 8.9 mmol/l, 56% of patients with fasting plasma glucose 8.9 to < 10 mmol/l and 47% of participants with fasting plasma glucose ≥ 10 mmol/l. Glucose-confirmed hypoglycaemia for these subgroups occurred in 34, 24 and 18%, respectively. Participants with 12-week fasting plasma glucose ≥ 10 mmol/l had significantly fewer symptomatic (*P* = 0.0149) and glucose-confirmed events compared with those with fasting plasma glucose < 8.9 mmol/l (*P* = 0.0145).

## Discussion

This pooled analysis of patient-level data confirmed the hypothesis that fasting plasma glucose measured early in the course of treatment can identify patients who are unlikely to achieve HbA_1c_ goals with glargine added to oral therapies and thus might benefit from re-evaluation of treatment options. Although the baseline fasting plasma glucose value provided little indication of whether a patient would attain ≤ 53 mmol/mol (7.0%) HbA_1c_ after 24 weeks of treatment, a single clinic-measured fasting plasma glucose value > 10 mmol/l between 6 and 12 weeks after starting glargine was associated with ∼25% chance of attaining that target. Also, a fasting plasma glucose value of ≥ 10 mmol/l after 12 weeks of treatment suggested approximately 15% likelihood of ending with HbA_1c_ > 75 mmol/mol (9.0%) and 7% likelihood of a final HbA_1c_ higher than 86 mmol/mol (10.0%). Not surprisingly, lower levels of fasting plasma glucose after 12 weeks of treatment with glargine were associated with more hypoglycaemia. However, the occurrence of severe hypoglycaemia was rare, even with intensive insulin therapy targeting fasting plasma glucose < 5.6 mmol/l. Thus, the occurrence of hypoglycaemia, when carefully managed, did not preclude intensification of insulin therapy.

These findings are potentially helpful despite the limitation inherent in their being based on only a single measurement of fasting plasma glucose for each patient at each time point. Collection of multiple self-measured values by the patient at home should provide a more substantial basis for evaluating the patient’s early response to insulin therapy. When, despite a systematic titration plan, glargine fails to reduce fasting plasma glucose below10 mmol/l within the first 12 weeks, potentially important problems may be suspected. These might include development of a new medical problem that interferes with insulin’s effectiveness, frequent omission of insulin injections, inability to make systematic decisions on dose–titration, mishandling of insulin or faulty injection technique, or eating prior to measurement of fasting plasma glucose either at home or in the provider’s office. If such medical or behavioural factors are not identified, the possibility of markedly elevated postprandial glucose values leading to wide daily variations of glucose should be considered, and addition of rapid-acting insulin or other prandial treatment may be indicated.

In conclusion, these analyses verify that attention to fasting plasma glucose changes in the first 12 weeks after starting treatment with glargine may allow earlier identification of patients unlikely to attain HbA_1c_ targets, leading to individualized attention and more timely and effective changes in management.
